# Revision Hip Arthroscopy in the Native Hip: A Review of Contemporary Evaluation and Treatment Options

**DOI:** 10.3389/fsurg.2021.662720

**Published:** 2021-07-05

**Authors:** Kyle N. Kunze, Reena J. Olsen, Spencer W. Sullivan, Benedict U. Nwachukwu

**Affiliations:** Hospital for Special Surgery, New York, NY, United States

**Keywords:** hip preservation, femoroacetabular impingement, clinical failure, revision arthroscopy, arthroplasty, outcomes

## Abstract

Hip arthroscopy is a reproducible and efficacious procedure for the treatment of femoroacetabular impingement syndrome (FAIS). Despite this efficacy, clinical failures are observed, clinical entities are challenging to treat, and revision hip arthroscopy may be required. The most common cause of symptom recurrence after a hip arthroscopy that leads to a revision arthroscopy is residual cam morphology as a result of inadequate femoral osteochondroplasty and restoration of head–neck offset, though several other revision etiologies including progressive chondral and labral pathologies also exist. In these cases, it is imperative to perform a comprehensive examination to identify the cause of a failed primary arthroscopy as to assess whether or not a revision hip arthroscopy procedure is indicated. When a secondary procedure is indicated, approaches may consist of revision labral repair, complete labral reconstruction, or labral augmentation depending on labral integrity. Gross instability or imaging-based evidence of microinstability may necessitate capsular augmentation or plication. If residual cam or pincer morphology is present, additional resection of the osseous abnormalities may be warranted. This review article discusses indications, the evaluation of patients with residual symptoms after primary hip arthroscopy, and the evaluation of outcomes following revision hip arthroscopy through an evidence-based discussion. We also present a case example of a revision hip arthroscopy procedure to highlight necessary intraoperative techniques during a revision hip arthroscopy.

## Introduction/Background

The prevalence of hip arthroscopy has increased as techniques for identifying and treating femoroacetabular impingement (FAI) continue to improve. This condition describes the abnormal contact of the femoral head–neck junction with the acetabulum and labral complex due to bony morphological abnormalities in the femoral head and/or acetabulum (1). Although widely successful with an overall low complication rate (4%), the clinical outcome is sometimes unsatisfactory (2). As the incidence of hip arthroscopy procedures performed annually continues to grow, so too does the incidence of patients who will require re-intervention and possible revision surgery. Therefore, it is imperative that hip arthroscopists must understand the presentation of patients with a failed hip arthroscopy and the etiologies of failure in order to identify such patients and treat them efficaciously.

The leading cause of clinical failure in hip arthroscopy is persistent FAI secondary to residual cam morphology, which may be combined with high-grade chondral damage and labral pathology (3, 4). Studies have estimated the average amount of time to be between 18 and 25.6 months between primary and revision surgeries (5, 6). Other etiologies of a failed primary hip arthroscopy include microinstability of the hip capsule, labral degeneration or re-tears, and progression to more severe grades of osteoarthritis, though other more rare etiologies exist. Revision candidates commonly present with missed or undertreated FAI, however, at varying rates. Philippon et al. (7) report the incidence of residual FAI in revision cases to be 95%, which is often a result of under-resection and over-resection leading to residual impingement or instability and leading to persistent or recurring symptoms postoperatively (8). Therefore, it is important to identify and fully treat FAI during the initial hip arthroscopy. Furthermore, it is important to identify the common failure mechanisms and to understand how to address them with secondary procedures.

The decision algorithm for revision hip arthroscopy can be complex and is affected by a wide range of factors. Current indications for revision arthroscopy and associated outcomes remain unclear and are a focus of this research study. The purpose of this study is to synthesize indications for revision hip arthroscopy following a failed primary arthroscopy using evidence to support these indications when available. Next, we describe the preferred surgical technique of the authors for a case of revision hip arthroscopy and then describe the other surgical approaches that exist. Finally, we will use an evidence-based discussion to describe the outcomes of revision hip arthroscopy. This information can facilitate preoperative discussion and planning between patients and their surgeons and can guide the expectations of patients about the procedure.

## Predictors, Etiologies, and Indications for Repeat Hip Arthroscopy

Ricciardi et al. (9) found that patients undergoing revision surgery were typically younger and female. Another study by West et al. (5) also confirmed younger age (<50 years) as a predictor of revision but did not see a significant difference in revision rates when looking at the gender of the patients. Some observed factors associated with revision are increased acetabular coverage (lateral center edge angle, LCEA, >33°), pistol grip/cam deformity before a primary arthroscopy, and unresolved high pistol grip deformity (10). Additionally, Shah et al. (11) identified predictors of failed arthroscopy necessitating revision, including small LCEA (moderate to severe hip dysplasia), larger Tonnis angle, ≤2 mm joint space, and a broken Shenton line.

There is a wide range of etiologies that may necessitate a revision hip arthroscopy that results in persistent symptoms and dysfunction (Table 1). Despite the etiology, the primary indication for revision hip arthroscopy is symptom recurrence. Residual FAI secondary to inadequate cam resection during the index procedure is the most common finding of a failed hip arthroscopy. Another common cause of symptomatic recurrence is microinstability of the hip capsule, though patients may also experience instability in the setting of cam over-resection and loss of the hip suction seal. Other etiologies that may necessitate revision hip arthroscopy include chondral wear, labral tears and calcifications, synovitis, adhesions, loose bodies, and instability (3, 4, 9). Full-thickness acetabular articular cartilage defect (FAACD) is chondral delamination that can cause pain and a catching sensation and, if left unaddressed during the index procedure, can contribute to loose bodies and progression of osteoarthritis (12). Open surgery may be required to address instability, dysplasia, or extra-articular impingement of the greater trochanter or subspine (9).

**Table 1:** Indications for revision hip arthroscopy.

Recurrent symptoms (hip pain, subjective instability, and dysfunction) reproducible on physical examination with at least ONE of the following:
- Alpha angle on AP or Dunn lateral 360 degrees or over-resection of more than 5% of the diameter of the femoral head on the Dunn view.
- Evidence of femoral head lucencies concerning for avascular necrosis
- Identifiable loose bodies on any imaging modality
- Evidence of labral calcification ± labral tear or fraying
- Any labral re-tear
- Subspine impingement
- Focal femoral head or acetabular chondral defects amenable to repair without Tonnis grade >1
- MRA evidence of capsular defects or laxity

Evaluation of the patient must be thorough to properly guide surgical decision-making. Importantly, another mechanism of failure consists of advanced cartilage pathology. Though patients may present with symptoms mimicking that they experienced prior to their index procedure, patient selection is a crucial aspect in this setting as more advanced stages of osteoarthritis should be treated with hip arthroplasty as to avoid a second failure (13). Therefore, a thorough understanding of the causes of the failed primary hip arthroscopy and which patients are appropriate candidates for a revision procedure is a key component of successful treatment.

## Clinical Evaluation

All patients with symptom recurrence warrant a thorough clinical examination in the postoperative setting. A thorough history may help the surgeon narrow the differential diagnosis. Pain is present in almost all patients and, therefore, non-specific; however, it is useful to prompt investigation into the underlying etiology as it may indicate labral re-tear. Infection should always be ruled out in this setting despite low likelihood, and the surgeon should order tests for complete blood count, erythrocyte sedimentation rate, and C-reactive protein. In patients with concomitant musculoskeletal pathologies, it is important to determine whether this pain is referred from the spine or is a result of intra-articular or extra-articular hip pathology. Patients with instability secondary to microinstability or previous capsulotomy without closure may report subluxation events where they believe their hip is “coming out of their socket” or have apprehension with certain movements that stress the iliofemoral ligament. Hip dysplasia, femoral anteversion >40°, connective tissue disorders, and previous traumatic hip injuries predispose individuals to post-arthroscopic hip instability (14). As most etiologies cause pain around the hip joint, physical examination and diagnostic imaging are crucial components of the evaluation.

Inspection of the previous portal incisions should be performed to rule out wound complications as this may point the surgeon to surgical site infection as the etiology of hip pain. Palpation of the pubic tubercle, greater trochanter, anterior superior iliac spine, and sacroiliac joints should be performed as these may point toward core muscle injury, bursitis, or other tendinopathies as the pain generator. Range of motion examination should be performed and compared with the opposite limb. A positive impingement sign can be clinically evaluated by performing the anterior impingement test by moving the hip in flexion, adduction, and internal rotation (FADIR) (15). Though rare, coxa saltans internal or external type may be identified with an audible snapping during the range of motion of the hip. Coxa saltans internal snapping is reproduced by passively moving the hip from a flexed and externally rotated position to an extended and internally rotated position. Patients with iatrogenic hip capsule instability may also demonstrate positive findings on axial distraction testing (16).

Repeat imaging of the symptomatic hip following a failed primary hip arthroscopic procedure is essential to understand the etiology. Imaging options to be used include anterior–posterior (AP) pelvis, false profile, and frog-leg or Dunn lateral radiographs, CT scans with or without three-dimensional (3D) reconstruction, and MRI. Plain radiographs of standing AP pelvis and Dunn view with 45° hip flexion can be used to identify residual cam and pincer impingement in addition to over-resection (1, 17). We do not believe that radiographic or CT evidence of borderline hip dysplasia should be a contraindication to revision hip arthroscopy as good outcomes have been reported in these populations (18, 19). CT imaging is suggested for assessing the abnormalities of acetabular and femoral versions that may contribute to the range of motion or impingement abnormalities, though this is not commonly obtained (17). A more useful application of CT in the revision setting is 3D reconstruction, which allows the surgeon to better plan their degree of chondroplasty in revision settings. The use of MRI may help identify labral re-tears, chondral damage, avascular necrosis, or stress fracture. This is especially important in the setting of labral re-tears that are irreparable due to calcification or lack of sufficient labrum with sufficient integrity as the surgeon may plan a labral reconstruction or augmentation for the revision. MR arthrogram (MRA) can be beneficial for visualizing the integrity of the hip capsule (20, 21). MRA evidence of capsular defects and instability on T1-weighted images include capsular scarring or capsular contraction, (2) anterior iliofemoral attrition or partial healing, (3) anterior iliofemoral separation and retraction, or (4) extracapsular dye extravasation due to gluteus minimus or gross capsular incompetency (21). McCormick et al. (21) suggest that capsular deficiency may be present in a high percentage of revision cases that are not primarily due to residual bony abnormalities. As such, capsular insufficiency should be considered when a patient presents with residual hip pain in the absence of obvious residual FAI.

An intra-articular hip steroid injection can be particularly important to confirm the surgical indication. A positive response to an intra-articular hip steroid injection is important for confirming the intra-articular nature of the problem. In patients with imaging and clinical examinations, pointing toward the need for revision surgery, but with a negative response to an intra-articular injection consideration of extra-articular sources of pain, should be considered. In particular, extra-articular sub-spine impingement, psoas tendinitis, lumbar spine pathology, and pelvic floor pain are often complicating diagnoses in patients with persistent pain after primary hip arthroscopy. The surgeon should have a lower threshold to perform revision hip arthroscopy in an expeditious manner when symptom recurrence in conjunction with positive imaging findings of a treatable etiology is present as to not predispose the patient to additional morbidity and joint degeneration.

## Surgical Techniques

The surgical preparation and hip arthroscopy setup are largely identical in all cases of revision hip arthroscopy. The major difference in revision hip arthroscopy is the procedures to be performed based on the history and clinical examination of the patient. The proceeding section briefly describes procedures commonly performed in revision hip arthroscopy.

### Labral Repair, Augmentation, and Reconstruction

Labral repair is indicated during revision hip arthroscopy when a patient presents with pain, and there is MRI evidence of a labral repair with sufficient tissues to repair. Debridement in the revision setting is uncommon as damaged tissues or re-tears are often not amenable to this treatment due to the quality of the tissue. In cases where the labrum is torn but is irreparable secondary to insufficient remaining tissue or tear size, a labral augmentation or complete reconstruction can be performed (22). Labral reconstruction can be segmental or circumferential depending on the extent and quality of labral degeneration. A tensor fascia lata allograft was first described to augment or reconstruct the labrum (23), though several graft options have since been used with good to excellent outcomes (24–26). We recommend the use of labral repair in the revision setting if the labrum is deemed reparable as we argue that this disrupts the anatomy and suction seal of the patient to a lesser extent than alternatives. A low threshold should be maintained to reconstruct the labrum if there is doubt as to the quality of remaining labral tissue. There is little evidence available as to whether a particular graft type is superior.

### Osteochondroplasty and Trimming for Residual Osseous Deformities

Acetabular rim trimming may be implicated for residual pincer morphology, while additional femoral osteochondroplasty may be implicated in the patient with symptom recurrence and evidence of residual cam morphology. It is imperative that preoperative radiographic indices of cam morphological dimensions can be made in order for the hip arthroscopist to appropriately plan the depth and extent of their resection, as over-resection can lead to instability and inferior outcomes as noted. These procedures are performed through the same approach and portals as used in a primary hip arthroscopy procedure. Intraoperative examination of the cartilaginous components of the femoral head and acetabular should be performed regardless of whether there is evidence of chondral lesions or delamination on preoperative imaging. If identified, focal chondral lesions can be addressed with microfracture, matrix-enhanced chondral implantation (27), or autologous chondrocyte implantation (28, 29). Though some studies have investigated the use of bone marrow aspirate concentration, platelet-rich plasma, and mesenchymal stem cells, the current evidence is of low quality (30).

### Capsular Management

Though we recommend complete capsular closure in all primary and revision hip arthroscopy cases, patients in whom the capsule was not closed or who have capsular and generalized ligamentous laxity should undergo complete capsular closure and/or plication. During revision hip arthroscopy, it is beneficial to establish identical portals in order to access the areas in which the capsule was previously violated in order to be able to successfully close them (i.e., the interportal capsulotomy sites). In patients with iatrogenic hip instability and without evidence of residual osseous abnormalities, it is appropriate to perform revision hip arthroscopy for capsular repair (16, 31).

### Extra-Articular Pathology

Snapping hip syndrome is infrequently an indication for revision hip arthroscopy, though patients may present with this pathology in conjunction with those described above. A recent study has described the use of an endoscopic iliotibial band release during hip arthroscopy for FAIS and coxa sultans external type with good short-term outcomes (32). There is a paucity of studies on iliopsoas tenotomy during hip arthroscopy, with reports of previous studies demonstrating that performing this additional procedure may predispose patients to inferior outcomes (33). As these studies have demonstrated the potential for worse outcomes after primary hip arthroscopy, we do not recommend performing these procedures in conjunction with revision hip arthroscopy for intra-articular or capsular etiologies.

## Outcomes

Research studies on outcomes and efficacy following revision hip arthroscopy are growing but are limited (3, 34, 35). Recent studies do show significant improvement in patient-reported outcome (PRO) following revision hip arthroscopy (34, 36). A meta-analysis by O'Connor et al. (37) reported a significant improvement in all PRO scores from before operation to the latest follow-up after revision, with the greatest average increase shown in the modified Harris Hip Score (mHHS) (+17.20) and the Hip Outcome Score–Activities of Daily Living (HOS-ADL) (+13.98), and a decrease in the visual analog scale for pain (VAS) (−3.16). Domb et al. (34) reported similar results from a study of 47 revision hip arthroscopies at a mean length of follow-up of 29 months, concluding a statistically significant improvement in each PRO measured: mHHS, HOS-ADL, HOS Sports Subscale (HOS-SS), VAS for pain, and the Non-arthritic Hip Score (NAHS). Positive pre-operative predictors for improvement in PROs are previous open surgery, FAI, symptomatic heterotopic ossification, and segmental labral defects (34). A pair-matched study comparing clinical outcomes after labral reconstruction vs. labral repair during revision arthroscopy was carried out by Perets et al. (38) and showed similar clinical improvement postoperatively and comparable complication rates. The authors concluded that both procedures are safe and effective labral repair treatment options during revision arthroscopy (38).

Despite many studies reporting statistically significant improvement in all clinical outcomes following revision hip arthroscopy surgery, these outcomes tend to be inferior when compared to patient outcomes following primary hip arthroscopy (17, 35). Larson et al. (35) matched cohorts of primary and revision arthroscopies and reported a significantly larger improvement in PROs for primary surgery patients in mHHS and VAS scores. It has been shown that after revision, improved PROs, high survivorship, and patient satisfaction are present at 2-year short-term clinical follow-up (36). Although research studies have shown that some positive results (outcome scores) following revision surgery have been reported to be less durable as compared to those following a primary arthroscopy, decreases in mHHS, satisfaction, HOS-ADL, and HOS-SS have been seen near the 3-year follow-up mark (34, 39).

Nwachukwu et al. (40) described values of minimal clinically important difference (MCID) and substantial clinical benefit (SCB) for patients undergoing revision hip arthroscopy to define meaningful improvement in outcomes. MCID is the smallest change in the outcome that can be appreciated by the patient, while SCB is a considerable change that a patient perceives as a substantial improvement. Considered, respectively, as the floor and upper threshold for clinical success, MCID and SCB values identified in this study on mHHS, HOS ADL, HOS-SS, and the international Hip Outcome Tool-33 (iHOT-33) were comparable to those values already defined for primary hip arthroscopy. Therefore, despite previously reported research studies showing that revision patients tend to report lower PROs than a primary arthroscopy cohort, when accounting for clinically meaningful improvement, these cohorts achieve comparable improvement in clinically significant outcomes. Additionally, revision patients presenting with residual impingement achieved MCID at a higher rate than patients with diagnoses other than FAI (40).

In some cases, patients may need a repeat revision hip surgery, i.e., a third hip arthroscopy. Despite the available studies demonstrating improvements in PROs and high survivorship after revision hip arthroscopy, there is a body of evidence reporting on second revision hip arthroscopy and conversion to hip arthroplasty (3, 6). In a comprehensive systematic review by Cvetanovich et al. (3), these reoperations occurred at an overall rate of 5% after an average of 14.9 months following a revision arthroscopy and up to 14.6% in the studies (6). Patients presenting with narrowing joint space and chondral damage during the evaluation of recurring symptoms after a primary arthroscopy are reported to have less improved outcomes and a greater likelihood of undergoing total hip arthroplasty (THA) following a revision (3). Mansor et al. (1) reported that cam over-resection on the Dunn view, that is >5% of the femoral head diameter, led to worse clinical outcomes following revision arthroscopy and lower survivorship with greater reports of conversion to THA. Due to the paucity of studies in reoperation rates following revision hip arthroscopy, research studies are limited in comparing patient outcomes of second revision surgery and primary THA.

## Conclusions

The leading cause of failure after primary hip arthroscopy leading to revision hip arthroscopy is residual cam morphology and symptom recurrence. The currently available studies suggest that patients undergoing revision hip arthroscopy can achieve good outcomes if indicated appropriately. Therefore, a thorough clinical examination and advanced imaging are imperative. Care should be taken to evaluate for chondral pathology and capsular incompetency in this setting. Failure to address these findings may result in inferior outcomes. The surgical technique should be tailored to the underlying cause for revision arthroscopy. Continued improvements in hip arthroscopy techniques and understanding of risk factors for failure will likely diminish the incidence of revision cases. This review article can be used to inform and guide identification, treatment, surgical decision-making, and expected outcomes of patients indicated for revision hip arthroscopy.

## Author Contributions

SS, RO, and BN contributed to conception and design of the study. RO, SS, and KK conducted literature reviews and data analysis. RO wrote the first draft of the manuscript. KK, SS, and BN wrote sections of the manuscript. KK performed all major revisions of the final manuscript and additional literature review necessary for publication. All authors contributed to manuscript revision, read, and approved the submitted version.

## Conflict of Interest

BN declares ownership interest in BICMD (founder) outside of the scope of the submitted manuscript. The remaining authors declare that the research was conducted in the absence of any commercial or financial relationships that could be construed as a potential conflict of interest.
